# Amide Proton Transfer Weighted Imaging Shows Differences in Multiple Sclerosis Lesions and White Matter Hyperintensities of Presumed Vascular Origin

**DOI:** 10.3389/fneur.2019.01307

**Published:** 2019-12-10

**Authors:** Elisabeth Sartoretti, Thomas Sartoretti, Michael Wyss, Anton S. Becker, Árpád Schwenk, Luuk van Smoorenburg, Arash Najafi, Christoph Binkert, Harriet C. Thoeny, Jinyuan Zhou, Shanshan Jiang, Nicole Graf, David Czell, Sabine Sartoretti-Schefer, Carolin Reischauer

**Affiliations:** ^1^Institute of Radiology, Kantonsspital Winterthur, Winterthur, Switzerland; ^2^Laboratory of Translational Nutrition Biology, Department of Health Sciences and Technology, ETH Zurich, Schwerzenbach, Switzerland; ^3^Philips Healthsystems, Zurich, Switzerland; ^4^Institute of Diagnostic and Interventional Radiology, University Hospital Zurich, University of Zurich, Zurich, Switzerland; ^5^Department of Radiology, Memorial Sloan Kettering Cancer Center, New York, NY, United States; ^6^Department of Medicine, University of Fribourg, Fribourg, Switzerland; ^7^Department of Radiology, HFR Fribourg-Hôpital Cantonal, Fribourg, Switzerland; ^8^Division of MR Research, Department of Radiology, Johns Hopkins University, Baltimore, MD, United States; ^9^Graf Biostatistics, Winterthur, Switzerland; ^10^Department of Neurology, Spital Linth, Uznach, Switzerland

**Keywords:** magnetic resonance imaging, amide proton transfer, molecular imaging, multiple sclerosis lesions, white matter lesions, CEST

## Abstract

**Objectives:** To assess the ability of 3D amide proton transfer weighted (APTw) imaging based on magnetization transfer analysis to discriminate between multiple sclerosis lesions (MSL) and white matter hyperintensities of presumed vascular origin (WMH) and to compare APTw signal intensity of healthy white matter (healthy WM) with APTw signal intensity of MSL and WHM.

**Materials and Methods:** A total of 27 patients (16 female, 11 males, mean age 39.6 years) with multiple sclerosis, 35 patients (17 females, 18 males, mean age 66.6 years) with small vessel disease (SVD) and 20 healthy young volunteers (9 females, 11 males, mean age 29 years) were included in the MSL, the WMH, and the healthy WM group. MSL and WMH were segmented on fluid attenuated inversion recovery (FLAIR) images underlaid onto APTw images. Histogram parameters (mean, median, 10th, 25th, 75th, 90th percentile) were calculated. Mean APTw signal intensity values in healthy WM were defined by “Region of interest” (ROI) measurements. Wilcoxon rank sum tests and receiver operating characteristics (ROC) curve analyses of clustered data were applied.

**Results:** All histogram parameters except the 75 and 90th percentile were significantly different between MSL and WMH (*p* = 0.018–*p* = 0.034). MSL presented with higher median values in all parameters. The histogram parameters offered only low diagnostic performance in discriminating between MSL and WMH. The 10th percentile yielded the highest diagnostic performance with an AUC of 0.6245 (95% CI: [0.532, 0.717]). Mean APTw signal intensity values of MSL were significantly higher than mean values of healthy WM (*p* = 0.005). The mean values of WMH did not differ significantly from the values of healthy WM (*p* = 0.345).

**Conclusions:** We found significant differences in APTw signal intensity, based on straightforward magnetization transfer analysis, between MSL and WMH and between MSL and healthy WM. Low AUC values from ROC analyses, however, suggest that it may be challenging to determine type of lesion with APTw imaging. More advanced analysis of the APT CEST signal may be helpful for further differentiation of MSL and WMH.

## Introduction

Differentiation between age related white matter hyperintensities of presumed vascular origin (WMH) in patients with small vessel disease (SVD) (1, 2) and demyelinating white matter lesions in patients with multiple sclerosis (MS) called multiple sclerosis lesions (MSL) can be difficult. MSL and WMH may have similar lesion morphology on MRI and may coexist in individual patients (3–5). Conventional magnetic resonance imaging (MRI) techniques such as T2 weighted (T2w) turbo/fast spine echo (TSE/FSE) images, proton density weighted (PDw) images, fluid attenuated inversion recovery (FLAIR) and double inversion recovery (DIR) images are highly sensitive to both WMH and MSL but do not provide evidence of the underlying etiology (3, 4, 6) because all these lesions are of similar morphological appearance on T2w MR images (1, 4, 5). Moreover, conventional MR imaging sequences do not provide any information on the histologically heterogeneous manifestation of MSL (7, 8) or WMH (2, 9–12).

As the diagnosis of MS is based on the number and location of white matter lesions that disseminate in space and time within the central nervous system (4, 6), WMH mimicking MSL can complicate the clinical diagnosis of MS (4). Thus, reliable imaging biomarkers that allow for a precise discrimination between these two entities are of great clinical interest (3–5).

Preclinical studies that address this issue suggest that co-registered T2^*^ and FLAIR images (4, 5) and proton magnetic resonance spectroscopy (^1^H-MRS) (1) may be useful in differentiating between MSL and WMH.

The recently introduced molecular imaging technique amide proton transfer weighted (APTw) imaging characterized by its high degree of rescan reproducibility, repeatability and reliability (13–16) has been successfully utilized in imaging of brain tumors and neurodegenerative diseases (14, 15, 17–19). While APTw imaging based on 3D fast spin echo sequences and magnetization transfer asymmetry (MTR_asym_) analysis may be confounded by relayed NOE effects, it offers an efficient way of characterizing tissue related changes in APT CEST effects with a 3D coverage of the brain. Preliminary reports have identified differences in APTw signal intensity in MS patients between MSL, normal appearing white matter (NAWM) and healthy white matter (WM) of control groups (18–20). While expanding into a more detailed analysis of purified changes in the APT effect (ΔAPT), the work by By et al. (18) shows differences in histogram analysis of MSL and NAWM based on asymmetry analysis. Furthermore, APTw techniques have also been used to image neurodegenerative diseases or ischemic lesions (21, 22).

Here, we hypothesize, that changes of the APT effect between MSL and WMH could be detected by 3D fast spin echo APTw imaging techniques and MTR_asym_ analysis, as implemented for clinical use in neuro oncology. Hence, we investigated the ability of APTw imaging to discriminate between MSL and WMH. For this purpose, both lesion types were identified, segmented and subsequently analyzed by comparing different histogram parameters. Receiver operating characteristic (ROC) curve analyses were performed to evaluate the diagnostic performance of these parameters. Ultimately, both lesion types were also compared to healthy WM in young volunteers not affected by any vascular or demyelinating disease process.

## Materials and Methods

### Study Subjects

While MSL and WMH (and their histological subtypes) cannot be distinguished with conventional MR imaging (3–5), lesions may be attributed to the MSL or WMH type (4, 5, 23) based on the symptoms and risk factors of the individual patient presenting with a certain lesion (4, 5). Thus, in line with previous similar studies patients (and thus ultimately lesions) were selected for the different subject groups depending on age (4–6), clinical symptoms/laboratory evaluation according to the most recent International Panel Criteria (6) and risk factors for SVD as outlined below (4, 5).

This study was carried out in accordance with the recommendations of the Cantonal Ethical Committee Zurich, Zurich, Switzerland with written informed consent from all subjects. All subjects gave written informed consent in accordance with the Declaration of Helsinki. The protocol was approved by the Cantonal Ethical Committee Zurich, Zurich, Switzerland with BASEC Number 2018-01275. The APTw data used in this study is provided in the Supplementary Material.

#### Subject Population for MS Lesions

A total of 27 patients (16 female, 11 male; age 18 to 59 years, mean age 39.6 years, median age: 36 years) with relapsing remitting multiple sclerosis or secondary progressive multiple sclerosis with an average duration of 9 ± 5 years were included in the study (4). Expanded disability status scale (EDSS) of patients was between 3 and 5 (18). The diagnosis of multiple sclerosis was confirmed in each patient according to the most recent International Panel Criteria (5, 6). Patients with risk factors for SVD (diabetes, arterial hypertension, smoking, hypercholesterolaemia, ischemic heart disease, peripheral vascular disease) (5) were excluded. Included patients had to present with at least one MSL.

#### Subject Population for WMH

A total of 35 patients (17 females, 18 males; age 49 to 89 years, mean age 66.6 years, median age: 68 years) routinely screened on cerebral MRI for the presence of cerebral metastases after diagnosis of carcinoma of various origin (especially bronchus and breast carcinoma) were included. Images from the most recent examination were used for the study. All 35 patients presented with at least two risk factors for SVD (arterial hypertension, diabetes, smoking, hypercholesterolemia, ischemic heart disease, peripheral vascular disease) (5). Patients presenting with cerebral metastases or with a clinical diagnosis of MS (or suspected MS) as defined by the most recent International Panel criteria (6) were excluded. Included patients had to present with at least one WMH.

#### Healthy Control Group for Healthy WM

To obtain APTw signal intensity values of healthy WM we opted for a separate healthy young control group as recommended by Dula et al. (19) rather than obtaining measurements from the NAWM of MS patients or patients presenting with WMH. NAWM on APTw imaging and on T2w maps is already known to be considerably affected by multiple sclerosis (18, 19, 24) and also by SVD (2). Therefore, the white matter cannot be considered healthy in these patients, but only appears normal on conventional MR images and is therefore called NAWM.

Overall, 20 healthy young volunteers (9 females, 11 males; age 19 to 37 years; mean age 29 years, median age: 28.5 years) were selected. Exclusion criteria for subjects were T2 hyperintense foci in gray and white matter on 2D T2 turbo spin echo (TSE) images and any other abnormalities. Subjects had to present without a current disease or a history of disease (such as a vascular or demyelinating disease). Specifically, subjects with cardiovascular risk factors (obesity, cardiac arrhythmia) or any risk factors for small vessel disease (diabetes, smoking, hypercholesterolaemia, ischemic heart disease, peripheral vascular disease) were excluded (5). As age is known to be a risk factor for SVD and microangiopathy (9) we specifically selected young subjects between the age of 18 to 40 years (4) as our aim was to measure APTw intensity values of normal healthy WM (19) without interference of WMH due to silent microangiopathy potentially influencing the APTw intensity values measured.

### MR Imaging

All subjects included were scanned on a 3T scanner (Achieva, Philips Healthcare, Best, the Netherlands) with an eight channel receive-only head coil array. For the evaluation of the MS patients the following sequences were performed as part of the MR protocol for routine evaluation: Transverse diffusion-weighted imaging (DWI), 3D double inversion recovery (DIR) sequence, precontrast 3D turbo field echo T1 weighted (T1w) sequence, postcontrast 3D FLAIR sequence and postcontrast 3D T1 black blood TSE or postcontrast 3D T1w m-Dixon turbo field echo (TFE) sequence. Furthermore, an APTw sequence and a 2D T2w TSE sequence were obtained.

In patients presenting with WMH the following sequences were acquired: Transverse DWI, precontrast 2Dw T1 fast field echo sequence (FFE) or 3D T1w black blood TSE sequence, postcontrast 3D FLAIR, and postcontrast 3D T1 black blood TSE +/– 3D T1w m-Dixon TFE. Furthermore, an APTw sequence and a 2D T2w TSE sequence were obtained.

For subjects in the healthy control group an APTw sequence and a 2D T2w TSE sequence were acquired.

The MR imaging parameters of the APTw-, the T2w and the 3D FLAIR sequence are shown in Table 1.

**Table 1:** Scan parameters of the APTw, 2D T2w TSE, and 3D FLAIR sequence.

	**3D APTw sequence**	**2D T2w TSE**	**3D FLAIR**
FOV	228 × 178 × 60 mm	230 × 230 × 165 mm	250 × 250 mm
Acquisition voxel	1.8 × 1.8 × 6.0 mm	0.6 × 0.6 × 3.5 mm	1.12 × 1.12 × 1.12 mm
Reconstruction voxel	0.9 × 0.9 × 3.85 mm	0.45 × 0.45 × 3.5 mm	1.04 × 1.04 × 0.56 mm
Reconstruction matrix	256 × 256	512 × 512	240 × 240
Slice thickness, Slice gap	3.85 mm, 0 mm	3.5 mm, 0.35 mm	3.85 mm, 0 mm (multiplanar reconstruction)
SENSE or Compressed SENSE factor	1.6 Sense	1.5 Sense	8 Compressed Sense
Scan mode	3D	2D	3D
TSE factor	174 with 3D TSE readout and 1,367 ms shot duration	30	182
Rest slabs	0	1	0
MultiVane percentage	–	160%	–
Flip angle (in degrees)	90	90	40
TR, TE, and TE equivalent	TR 5,800 to 5,864 ms TE 7.8 to 8.3 ms	TR 4,000 ms TE 120 ms	4,800, 278, and 120 ms
Inversion time TI	–	–	1,650 ms
Fat suppression	SPIR	SPIR	SPIR
APTw	Saturation B_1rms:_ 2 μT Saturation duration: 2 s	–	–
Number of acquisitions NSA	1	1	2
Scan duration	03 min 42 s	03 min	04 min 43 s

#### APTw Imaging

A slightly modified version of the clinically approved APTw sequence by Philips Healthcare (13, 25) was used (3.85 mm instead of 6 mm slice thickness) because lesions can be quite small and thus a smaller slice thickness decreases the influence of partial volume effects. The APTw sequence was scanned in transverse oblique orientation parallel to the intercommissural line. Sixteen slices with a slice thickness of 3.85 mm were acquired. The first slice was centered at the inferior border connecting the rostrum and the splenium of the corpus callosum.

To generate APTw imaging contrast, magnetization transfer ratio asymmetry (MTR_asym_) was calculated according to the following formula:

$$\begin{array}{l} MTR_{asym}\;\left(\%\right)=\;\frac{\left(S_{-\Delta \omega }-S_{\Delta \omega }\right)\;}{S_{0}} \end{array}$$

S_−Δω_ and S_Δω_ correspond to the water signal at negative and positive frequency offset. S_0_ is the signal without radiofrequency saturation (13, 25). MTR_asym_ is based on the acquisition of a Z-spectrum, where multiple water signal levels are measured as a function of different frequency offsets (Δω). The water signal saturation is calculated as a function of the saturation frequency on this spectrum (25).

For the Z-spectrum, nine image volumes at seven different frequency offsets (± 3.1 ppm, ± 3.5 ppm, ± 3.9 ppm, and −1,560 ppm) were acquired (13, 25). A B_0_ map derived from three acquisitions at +3.5 ppm with slightly different echo shifts using an mDIXON algorithm was used for a voxel-by-voxel B_0_ correction (13, 25). B_1_ shimming was performed for each scan thus allowing for B_1_ inhomogeneity correction as described in detail by Togao et al. (13). APTw intensity values in this paper always represent MTR_asym_ values at 3.5 ppm offset frequency (Δω) quantified in % water signal intensity (13, 25). In case of intravenous injection of Gadolinium, the APTw image acquisition was always performed before administration of Gadolinium.

### Lesion and Healthy WM Selection

All non-confluent lesions (3) in the periventricular, deep, subcortical and juxtacortical white matter in frontal and parietal lobe, with the centrum semiovale included, both in the MSL and WMH subject group, were chosen for analysis based on the position of the APTw sequence that covered brain areas from corpus callosum to vertex. FLAIR hyperintense linear periventricular rims were excluded. Lesions encompassing <10 voxels were excluded to ensure a minimal lesion size of 3 mm (3, 5). A total number of 346 MSL (median number of voxels in lesions: 52; range 10–906, 45.3% from right hemisphere, 54.6% from left hemisphere) and 220 WMH (median number of voxels in lesions: 50; range 10–1347, 57.7% from right hemisphere, 42.3% from left hemisphere) were included in the analysis.

MSL were only selected from the MS subject group while WMH were only selected from the WMH subject category.

MSL of the MS patient group were selected according to the revised McDonald criteria 2017 (6) as discrete areas with FLAIR, DIR and T2w TSE hyperintensity, T1w iso- to hypointensity and iso- to slight DWI hyperintensity (6).

WMH of the WMH subject group were identified according to MR imaging criteria as discrete areas with FLAIR and T2w TSE hyperintensity, T1w iso- to hypointensity and DWI isointensity (2, 5, 9–11).

**Figure 1:**
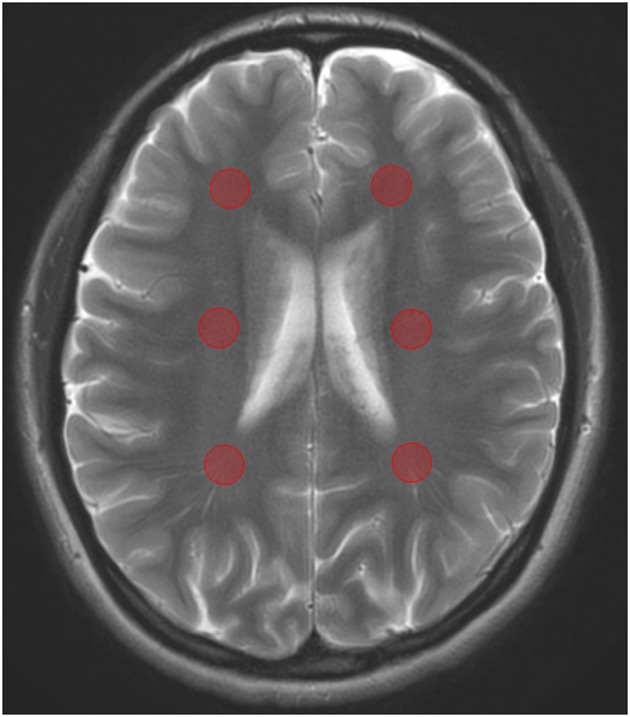
T2w image with 0% APT image overlay. ROIs for measurements in normal white matter in healthy volunteers are performed bilaterally in the frontal white matter, in the centrum semiovale and in the parietal white matter. ROI size varied between 80 and 90 mm^2^ in different healthy volunteers.

To obtain values of healthy WM, ROI measurements were performed bilaterally in the white matter of the frontal lobe, the parietal lobe, and the centrum semiovale in each subject (thus totaling 6 measurements per subject and thus 120 measurements in total) of the healthy control group (Figure 1).

### Postprocessing and Image Analysis

To analyze MSL or WMH, the transverse reconstructions of the 3D FLAIR sequence, the APTw transverse images and the T2w images were transferred to an independent workstation “Intellispace Discovery, Version 2.0” (Philips Healthcare, Best, the Netherlands), where all the sequences were coregistered. With the help of a software program named “research oncology suite” the selected lesion was first outlined manually on the FLAIR slice (3) where the lesion showed its maximum diameter and verified on the T2w image. Second the program automatically segmented the volume of the entire lesion on all adjacent slices with a combined threshold and edge detection function. Correct segmentation was adjusted manually in case of misalignment. This segmented lesion volume was transferred and overlaid to the APTw sequence and the values of the APTw signal intensity were derived voxel-wise from this volume of interest. The data was then exported as a simple text file and used to create histograms. Ultimately, histogram parameters (mean, median, 10th percentile, 25th percentile, 75th percentile, 90th percentile) were calculated in all extracted WMH and MSL.

To obtain values of healthy WM from the healthy control group, APTw images were co-registered and overlaid with the geometrically identically acquired T2w TSE images on a dedicated workstation “IntelliSpace Portal” version 8 (Philips Healthcare, Best, the Netherlands). After fusion and co-registration it was possible to switch from the pure T2w TSE image, to the fused T2w TSE and APTw image (both each 50% contributing) to the pure APTw color coded image. ROIs were defined on the underlying T2w TSE image and the APTw intensity value was measured on the corresponding APTw image. The ROI size depended on the size of the anatomical structure and thus the size of the ROI was chosen based on the size of the anatomical region. Round and oval ROI sizes were used based on the anatomical configuration of the respective anatomical structure. ROIs for each white matter region were always copied (and adjusted in their position) from one hemisphere to the other to ensure that identical ROIs were used in bilateral measurements. As proposed in a similar study (26) mean APTw signal intensity values (rather than all histogram parameters) were then calculated for each ROI.

Lesion selection and measurements were performed by two trainees (E.S. and T.S.) and controlled by a neuroradiologist with 30 years of experience (S.S). In case of disagreement, a second neuroradiologist with 5 years of experience (A.S) was additionally consulted and the selection or measurement was discussed and adjusted until consensus was reached.

### Statistical Analysis

To compare the age distributions between the three subject groups, Wilcoxon rank sum tests were applied. Gender distributions were compared with the chi-square test.

For each patient, the mean for all histogram parameters (mean, median, 10th percentile, 25th percentile, 75th percentile, 90th percentile) was calculated and compared with a Wilcoxon rank sum test between MSL and WMH. The *p*-values were corrected with the Benjamini & Hochberg procedure with a false discovery rate of 0.10. The Wilcoxon rank sum test was utilized to compare histogram parameter mean of MSL and WMH with histogram parameter mean of healthy WM.

The diagnostic performance of different histogram parameters for differentiation of MSL from WMH was evaluated by means of ROC curve analyses. To adjust for possible correlations of APTw data within patients, clustering was accounted for (27). The Youden index and corresponding sensitivity and specificity were computed for all histogram parameters. *P*-values < 0.05 were considered significant.

## Results

### Comparison of Age and Gender Distributions Between Subject Groups

Patients within the WMH category (median: 68 years) were significantly older than MS patients (median: 36 years) (*p* < 0.001), which is in line with the results of a recent similar study (5). WMH are observed more frequently in elderly individuals and can be more often attributed to microangiopathic etiology in these individuals (9) and therefore older people represent a more representative comparator group for this kind of study (5). As expected, the control group for healthy WM (median: 28.5 years) was significantly younger than the MSL patient group (median: 36 years) (*p* = 0.004) and the WMH patient group (*p* < 0.001). There was no difference in the gender distributions between the three subject groups (*p* = 0.58).

### Comparison of MSL and WMH

**Figure 2:**
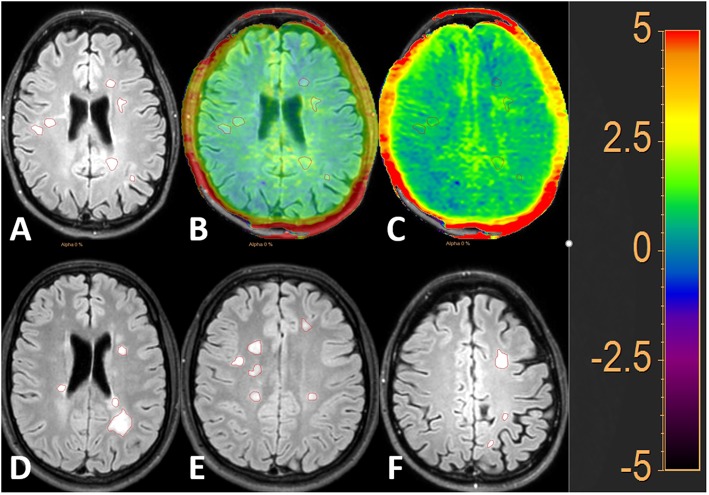
FLAIR image with 50% and 100% APTw image overlay. MS lesions (MSL) are outlined in red. The image to the left shows the lesions on FLAIR **(A)**, the middle image shows the FLAIR image with 50% APTw overlay **(B)** and the image to the right shows the FLAIR image with 100% APTw overlay **(C)**. Additional examples of the lesion selection are given in **(D–F)**.

**Figure 3:**
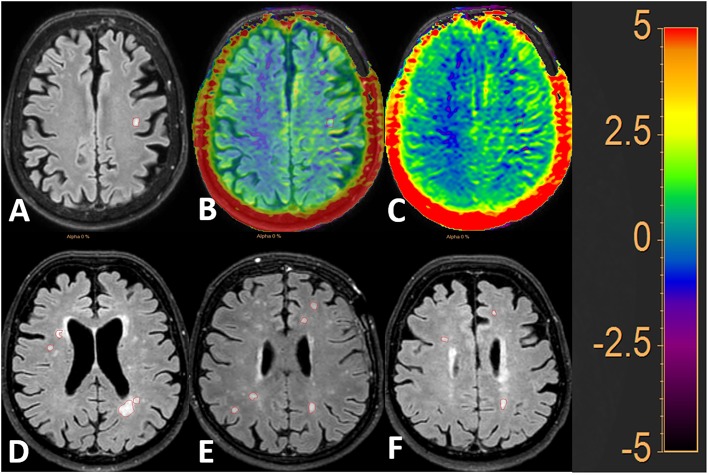
FLAIR image with 50 and 100% APTw image overlay. White matter hyperintensities of presumed vascular origin (WMH) are outlined in red. The image to the left shows the lesions on FLAIR **(A)**, the middle image shows the FLAIR image with 50% APTw overlay **(B)** and the image to the right shows the FLAIR image with 100% APTw overlay **(C)**. Additional examples of the lesion selection are given in **(D–F)**.

Two exemplary cases of MSL and WMH lesion selection and segmentation on FLAIR and APT are shown in Figures 2, 3.

**Figure 4:**
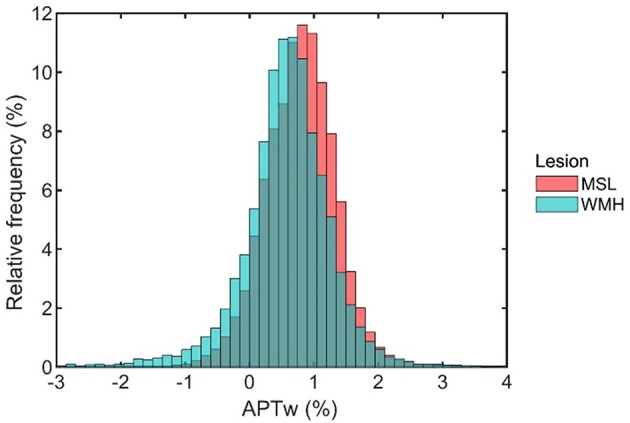
Histogram profiles encompassing all voxels obtained from MS lesions (MSL) and white matter hyperintensities of presumed vascular origin (WMH).

Histogram profiles of MSL and WMH are depicted in Figure 4. The overall histogram profile of MSL was shifted toward slightly higher APTw intensity values (thus more to the right side in Figure 4) than the overall histogram profile of WMH.

### Comparison of Histogram Parameters Between MSL and WMH

**Figure 5:**
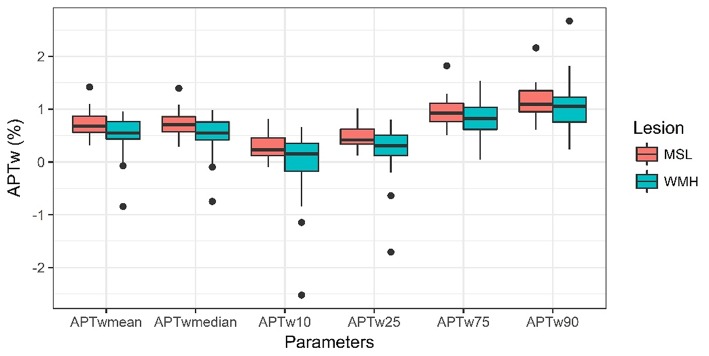
Comparison of histogram parameters (mean, median, 10th percentile, 25th percentile, 75th percentile and 90th percentile) of APTw data from MS lesions (MSL) (*n* = 27) and white matter hyperintensities of presumed vascular origin (WMH) (*n* = 35) that were averaged for each patient. The line in the box shows the median, the lower and upper hinges correspond to the first and third quartiles, the upper/lower whisker extends from the hinge to the largest/smallest value no further than 1.5 * IQR from the hinge.

Table 2 shows the comparisons of histogram parameters and the data is additionally visualized in Figure 5. In summary, the mean, median, 10th percentile and 25th percentile APTw intensity values of MSL were significantly higher than the intensity values of WMH (*p* = 0.034, *p* = 0.018, *p* = 0.026, *p* = 0.024). No significant difference was detected between 75th percentile and 90th percentile values (*p* = 0.104, *p* = 0.247).

**Table 2:** Comparison of histogram parameters that were averaged for each patient.

**APTw intensity histogram parameters (%)**	**MSL** **mean ± SD;** **median; [IQR]**	**WMH** **mean ± SD;** **median; [IQR]**	**Raw *p*-value**
Mean	0.72 ± 0.24; 0.68; [0.56,0.87]	0.52 ± 0.35; 0.55; [0.44,0.77]	0.034^*^
Median	0.72 ± 0.24; 0.71; [0.57,0.86]	0.52 ± 0.34; 0.55; [0.42,0.76]	0.018^*^
10th percentile	0.3 ± 0.24; 0.23; [0.12,0.46];	−0.01 ± 0.6; 0.15; [−0.18,0.35]	0.026^*^
25th percentile	0.49 ± 0.23; 0.42; [0.34,0.62]	0.25 ± 0.46; 0.30; [0.12,0.51]	0.024^*^
75th percentile	0.95 ± 0.28; 0.93; [0.77,1.11]	0.8 ± 0.32; 0.82; [0.62,1.04]	0.104
90th percentile	1.14 ± 0.33; 1.10; [0.95,1.35]	1.05 ± 0.43; 1.05; [0.76,1.23]	0.247

Mean ± standard deviation (SD), median and interquartile range (IQR) are shown. Significant p values using the Benjamini & Hochberg procedure with a false discovery rate of 0.10 are marked with a star (^*^).

### Diagnostic Performance of Histogram Parameters of MSL and WMH

**Figure 6:**
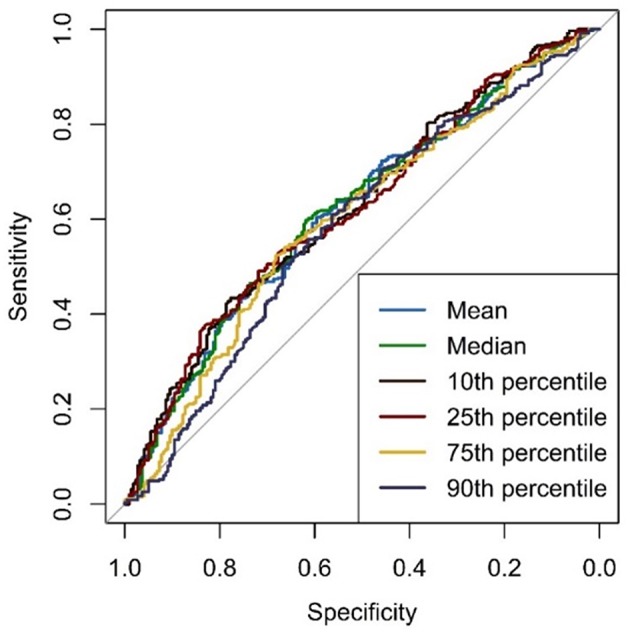
ROC curves of APTw histogram parameters from MS lesions (MSL) and white matter hyperintensities of presumed vascular origin (WMH).

The diagnostic performance obtained from ROC curve analysis is shown in Table 3. ROC curves are depicted in Figure 6. The parameters analyzed (mean, median, 10th, 25th, 75th, and 90th percentile) only offered low diagnostic performance (range of AUC: 0.5835–0.6245). The highest diagnostic performance (AUC = 0.6245, 95% CI: [0.5323, 0.7167]) was achieved with the 10th percentile APTw signal intensity values.

**Table 3:** AUC and 95% confidence interval (95% CI), cutoff value (Youden index), sensitivity, specificity, and accuracy for each histogram parameter from MS lesions (MSL) and white matter hyperintensities of presumed vascular origin (WMH).

**APTw intensity histogram parameters (%)**	**AUC; [95% CI]**	**Cutoff value (%)**	**Sensitivity (%)**	**Specificity (%)**	**Accuracy (%)**
Mean	0.618 ^*^; [0.529, 0.708]	0.654	60.4	59.5	60
Median	0.620 ^*^; [0.532, 0.709]	0.660	59.8	61.8	60.6
10th percentile	0.625 ^*^; [0.532, 0.717]	0.448	43.4	77.7	56.7
25th percentile	0.621 ^*^; [0.534, 0.709]	0.570	49.1	71.8	58
75th percentile	0.600 ^*^; [0.506, 0.694]	0.983	52.3	68.2	58.5
90th percentile	0.584; [0.482, 0.686]	1.066	61	56.4	59.2

Significant AUC values are marked with a star (^*^).

### Comparison of APTw Intensity Between Healthy WM and MSL and Healthy WM and WMH

**Figure 7:**
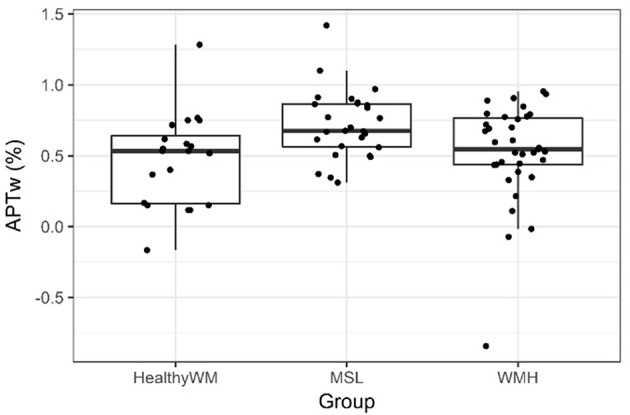
Boxplots with jittered individual data points depicting mean APTw signal intensity values of healthy white matter (HealthyWM) (*n* = 20), MS lesions (MSL) (*n* = 27), and white matter hyperintensities of presumed vascular origin (WMH) (*n* = 35) that were averaged for each patient/proband. The line in the box shows the median, the lower and upper hinges correspond to the first and third quartiles, the upper/lower whisker extends from the hinge to the largest/smallest value no further than 1.5 * IQR from the hinge.

As explained in the methods section, the mean APTw signal intensity value [rather than all histogram parameters (26)] was calculated for each ROI of healthy WM and compared with the mean APTw signal intensity of MSL and WMH. The results indicated that the APTw signal intensity of MSL (mean ± SD: 0.72 ± 0.24%; median: 0.68%, IQR; [0.56, 0.78%]) differed significantly from the APTw intensity of healthy WM (mean ± SD: 0.47 ± 0.32%; median: 0.53%, IQR; [0.16, 0.64%]) (*p* = 0.005). However, the APTw signal intensity of WMH (mean ± SD: 0.52 ± 0.35%; median: 0.55%, IQR; [0.44, 0.77%]) did not differ significantly from the signal intensity of healthy WM (mean ± SD: 0.47 ± 0.32%; median: 0.53%, IQR; [0.16, 0.64%]) (*p* = 0.345). The data is visualized in Figure 7.

## Discussion

In this study a systematic analysis of APTw signal intensity values in MSL and WMH was performed. Lesions were carefully selected, segmented and analyzed with histogram parameters. Histogram analysis has been shown to yield a high degree of inter-observer reproducibility and is an established method to analyze APTw signal intensity values (26, 28). APTw imaging, characterized by a high degree of scan rescan reproducibility, repeatability and reliability (13–16), was performed with a slightly modified version (modified slice thickness) of the only clinically approved APTw sequence currently available commercially by Philips Healthcare® (13, 25). A voxel-by-voxel B_0_ and B_1_ correction was implemented during APTw imaging (13, 25).

Our results showed that all histogram parameters except the 75th and 90th percentile were significantly different between MSL and WMH. The histogram parameters analyzed offered only low diagnostic performance in discriminating between MSL and WMH. The 10th percentile yielded the highest diagnostic performance with an AUC of 0.6245. Hence while MSL and WMH may show statistically significant quantitative differences, it is questionable whether the small differences in the APTw signal intensity can be used clinically for differentiation of individual lesions. Addition of other techniques and sequences to histogram parameters as for example co-registered T2^*^/FLAIR images (as described in previous preclinical studies) may increase diagnostic performance (4, 5). Lastly, we found that the mean APTw signal intensity values of MSL were significantly higher than the values of WMH and of healthy WM. However, the values of WMH did not differ significantly from the values of healthy WM in young volunteers.

To our knowledge, differences in APTw signal intensity values between MSL and WMH have not yet been published. However, previous studies have investigated white matter changes of APTw signal intensity values in the cervical spinal cord and brain of MS patients (18, 19). Great variability in results between these studies were observed (20).

Both studies found significant differences between NAWM and healthy WM (18, 19) and thus, in order to obtain reliable values for healthy WM, inclusion of a healthy control group was recommended for future studies (19).

In the cervical spinal cord of MS patients no significant difference between MSL and healthy WM was reported (18). However, differences between cerebral MSL and healthy WM were observed in MS patients (19). Specifically, higher APTw signal intensity values in some MSL compared to healthy WM was observed, but there was also variability between individual MSL (19).

Similar to these results we observed significantly higher APTw signal intensity in MSL than in healthy WM.

In theory, the APTw signal is caused by two major sources, namely the intracellular water-exchangeable amide proton content in the cytoplasm and the base-catalyzed exchange rate at physiological pH range (21). However, it should be acknowledged that the APTw signal may be contaminated by a variety of other technical factors (18, 29–34). It is known that water longitudinal relaxation time (T_1_) influences the APTw signal. The signal may either be influenced linearly or in a complex manner by T_1_ effects (T_1_ recovery and T_1_ - related saturation) depending on the level of direct water saturation effects, the field strengths of the MR scanner, irradiation power and whether non-steady-state or steady-state acquisitions are performed. Additionally, semi-solid magnetization transfer (MT) and other nearby CEST and relayed nuclear Overhauser enhancement (rNOE) saturation transfer effects have been shown to affect the APTw signal. Lastly an imperfect distribution of the irradiation power across the brain can trigger B1 effects which may also impact APTw intensity values (34).

It has been hypothesized that the increase in APTw signal intensity values in MSL in MS patients may be caused by changes in the intracellular amide proton content. Increased protein accumulation and concentration as seen in activated microglia surrounding chronic active MSL or increased protein degradation during axonal damage with secondary higher concentrations of mobile peptides may be responsible for an increase in APTw signal intensity (18, 20, 21).

The differences in results from these studies may be attributed to different anatomies examined, different APTw sequences and parameters utilized, different field strengths (7T vs. 3T) and by small sample sizes of patients included in the previous studies (18, 19). Furthermore, it has been hypothesized that heterogeneity of results may also stem from histological differences between MSL both in and between patients (18, 19).

MSL are histologically classified as preactive, active, mixed chronic-inactive/active or chronic-inactive depending on the degree of microglia activation, immune response and demyelination (7, 8).

In our study as well as in similar recently published studies (3–5, 23) the various known histological subtypes of MSL could not be differentiated because conventional MR imaging techniques are not able to differentiate the MSL according to their histologic differences (7, 8) and therefore APTw signal intensity values represent a mixture of APTw signal intensity values of histologically different MSL.

Concerning the APTw signal of WMH, pH may play a decisive role. In ischemic lesions, the exchange rate of amide protons is affected due to its sensitivity by pH changes (21, 22). In acute ischemic stroke, elevated APTw signal intensity values are attributed to intracellular acidosis (22, 35–38). This intracellular tissue acidification will alleviate in the follow up if clinical symptom improvement occurs after ischemic stroke (with or without treatment) (38, 39). Thus, patients usually present with a gradually increasing and normalizing APTw signal intensity over time after onset of an ischemic stroke despite persisting severe ischemic changes on FLAIR images (38, 39). This normalization occurs in the subacute stage after 4 to 7 days after acute ischemic stroke where APTw signal intensity values were identical in ischemic tissue and in normal contralateral white matter (39). In this study we report similar APTw signal intensity values in WMH, representing chronic lesions, and in healthy WM of young healthy volunteers thus confirming these previous observations.

WMH also do not represent a histologically homogenous group of lesions. This is reflected even in their appearance on T2w- and FLAIR images, where these lesions present with various degrees of FLAIR hyperintensity. This matter of fact most likely depends on the severity of the SVD (2, 9–11) and the exact disease stage (9, 10, 12, 40–42). WMH may present with impaired blood brain barrier, loss of oligodendrocytes and reduced density of glia, vacuolation and axonal damage and impaired white matter integrity as reported on diffusion tensor imaging (2, 9, 10, 12, 43–45). However, as in MSL, conventional MRI does not allow for a differentiation of histologically different WMH (4, 5).

As for the significant differences observed between MSL and WMH, varying degrees of the two major sources contributing to the APTw signal, namely the intracellular water-exchangeable amide proton content in MSL and the exchange rate of amide protons influenced by the pH value in the tissue in WMH may be responsible. However, these differences in APTw signal intensity values seem too small to allow for a reliable differentiation of individual lesions. A higher diagnostic performance may be achieved by combining APTw histogram parameters with other metrics and MRI technologies (especially MR sequences) that may facilitate differentiation of MSL and WMH. Specifically preclinical studies have shown that morphological features of lesions as T2^*^/FLAIR characteristics may be useful for differentiation of lesions (3–5) and thus may offer additional value when combined with APTw histogram parameters. Lastly, more advanced analysis of the APT CEST signal may also be helpful for further differentiation of MSL and WMH.

Our study has several limitations:
Firstly, it is possible that an individual lesion in the white matter of an elderly MS patient was wrongly allocated to the MSL group despite careful lesion and patient selection. Patients (and lesions) were selected based on criteria described in previous *in vivo* studies for MSL and WMH (2–6, 9–11). To further increase accuracy of lesion selection, biopsy samples or post mortem investigations would be necessary.
Secondly, due to the selection criteria of subjects in this study, the three subject groups had significantly different age distributions. Different age distributions (as in the case of MSL subject group vs. WMH subject group) in subject groups enable a more accurate selection of lesions (5). Furthermore, young volunteers were recruited for the healthy control group in order to minimize the prevalence of asymptomatic WMH. Specifically, it is known that even in individuals between 45 and 55 years of age, more than 50% of patients are affected by WMH (4). Nonetheless, it is unclear how age affects APTw signal intensity values of WM and this may have influenced our results. Thus, future studies should evaluate the impact of age on APTw signal intensity values in different brain regions, both in brains affected by disease and healthy brains.
Thirdly, MSL and WMH (and thus healthy WM values) were only selected from three anatomical regions, namely the frontal and parietal white matter and the centrum semiovale due to geometrical constraints of the APTw sequence.

## Conclusion and Outlook

MSL histogram parameters differed significantly from WMH histogram parameters. Furthermore, APTw signal intensity values in MSL differed significantly from the values in healthy WM while APTw signal intensity values in WMH did not differ from the values in healthy WM. However, due to the overall small differences, histogram parameters analyzed yielded only a low degree of diagnostic performance for differentiation between MSL and WMH. A higher diagnostic performance may be achieved by combining APTw histogram parameters with other metrics and MR technologies (especially other MR sequences) and morphological features of lesions. Specifically the correlation of imaging features with histological data may improve lesion selection thus potentially reducing the heterogeneity of lesions included in future studies. Furthermore, other histogram parameters (such as kurtosis and skewness) may also be useful to distinguish MSL from WMH. This should be investigated in further studies.

## Data Availability Statement

All data generated for this study are included in the article/Supplementary Material.

## Author Contributions

ES, TS, CR, AB, SS-S, MW, JZ, and SJ designed the study and interpreted the results. TS, SS-S, MW, LS, ÁS, and AN performed the experiments. AB, JZ, MW, TS, and ES analyzed the data. TS, SS-S, AB, and MW wrote the paper. CB, HT, DC, and CR provided technical advice. NG and TS conducted the statistical analysis. All coauthors contributed constructively to the manuscript.

### Conflict of Interest

MW is a part time employee of Philips Healthcare Switzerland. The remaining authors declare that the research was conducted in the absence of any commercial or financial relationships that could be construed as a potential conflict of interest.

## Supplementary Material

Data Sheet 1 APTw values used in this study.

## Abbreviations

MS: multiple sclerosis
MSL: multiple sclerosis lesions
NAWM: normal appearing white matter
Healthy WM: healthy white matter
ROC: receiver operating characteristic
SD: standard deviation
SVD: small vessel disease
WM: white matter
WMH: white matter hyperintensities
FLAIR: fluid attenuated inversion recovery
T2w: T2 weighted
TSE: turbo spin echo
DWI: diffusion weighted imaging
MRI: magnetic resonance imaging
FSE: fast spin echo
PDw: proton density weighted
T1w: T1 weighted
SPIR: spectral presaturation inversion recovery
SENSE: sensitivity encoding
MTR_asym_: magnetization transfer ratio asymmetry
IQR: interquartile range.

## References

[1] Kapeller P Ropele S Enzinger C Lahousen T Strasser-Fuchs S Schmidt R. Discrimination of white matter lesions and multiple sclerosis plaques by short echo quantitative 1H-magnetic resonance spectroscopy. J Neurol. (2005) 252:1229–34. 10.1007/s00415-005-0847-3 15895306

[2] Shi Y Wardlaw JM. Update on cerebral small vessel disease: a dynamic whole-brain disease. Stroke Vasc Neurol. (2016) 1:e000035. 10.1136/svn-2016-000035 28959468 PMC5435198

[3] Hosseini Z Matusinec J Rudko DA Liu J Kwan BYM Saleh Fi Morphology-specific discrimination between ms white matter lesions and benign white matter hyperintensities using ultra-high-field MRI. AJNR Am J Neuroradiol. (2018) 391:1473–9. 10.3174/ajnr.A5705 PMC7410534 29930096

[4] Kilsdonk ID Wattjes MP Lopez-Soriano A Kuijer JPA de Jong MC de Graaf WL. Improved differentiation between MS and vascular brain lesions using FLAIR^*^ at 7 Tesla. Eur Radiol. (2014) 24:841–9. 10.1007/s00330-013-3080-y 24317461

[5] Campion T Smith RJP Altmann DR Brito GC Turner BP Evanson J. FLAIR^*^ to visualize veins in white matter lesions: a new tool for the diagnosis of multiple sclerosis? Eur Radiol. (2017) 27:4257–63. 10.1007/s00330-017-4822-z 28409356 PMC5579202

[6] Thompson AJ Banwell BL Barkhof F Carroll W Coetzee T Comi G. Diagnosis of multiple sclerosis: 2017 revisions of the McDonald criteria. Lancet Neurol. (2018) 17:162–73. 10.1016/S1474-4422(17)30470-2 29275977

[7] Jonkman LE Lopez Soriano A Amor S Barkhof F van der Valk P Vrenken H. Can MS lesion stages be distinguished with MRI? A postmortem MRI and histopathology study. J Neurol. (2015) 262:1074–80. 10.1007/s00415-015-7689-4 25761376 PMC4412507

[8] Kuhlmann T Ludwin S Prat A Antel J Brück W Lassmann H. An updated histological classification system for multiple sclerosis lesions. Acta Neuropathol. (2017) 133:13–24. 10.1007/s00401-016-1653-y 27988845

[9] Wardlaw JM Valdés Hernández MC Muñoz-Maniega S. What are white matter hyperintensities made of? Relevance to vascular cognitive impairment. J Am Heart Assoc. (2015) 4:001140. 10.1161/JAHA.114.001140 26104658 PMC4599520

[10] Wardlaw JM Smith C Dichgans M. Mechanisms of sporadic cerebral small vessel disease: insights from neuroimaging. Lancet Neurol. (2013) 12:483–97. 10.1016/S1474-4422(13)70060-7 23602162 PMC3836247

[11] Martorella Medrano S Cuadrado Blázque M García Figueredo B González Ortiz S Capellades Font J Update in Radiology. Hyperintense punctiform images in the white matter: a diagnostic approach. Radiología. (2012) 54:321–35. 10.1016/j.rxeng.2011.09.001 22284561

[12] Schmidt R Schmidt H Haybaeck J Loitfelder M Weis S Cavalieri M. Heterogeneity in age-related white matter changes. Acta Neuropathol. (2011) 122:171–85. 10.1007/s00401-011-0851-x 21706175

[13] Togao O Hiwatashi A Keupp J Yamashita K Kikuchi K Yoshiura T. Scan-rescan reproducibility of parallel transmission based amide proton transfer imaging of brain tumors. J Magn Reson Imag. (2015) 42:1346–53. 10.1002/jmri.24895 25828573

[14] Li C Peng S Wang R Chen H Su W Zhao X. Chemical exchange saturation transfer MR imaging of Parkinson's disease at 3 Tesla. Eur Radiol. (2014) 24:2631–9. 10.1007/s00330-014-3241-7 25038850 PMC4471479

[15] Li C Chen M Zhao X Wang R Chen H Su W Chemical exchange saturation transfer MRI signal loss of the substantia nigra as an imaging biomarker to evaluate the diagnosis and severity of Parkinson’s disease. Front Neurosci. (2017) 31:489 10.3389/fnins.2017.00489 PMC5583514 28912676

[16] Khlebnikov V Windschuh J Siero JC Zaiss M Luijten PR Klomp DW. On the transmit field inhomogeneity correction of relaxation-compensated amide and NOE CEST effects at 7 T. NMR Biomed. (2017) 30:e3687. 10.1002/nbm.3687 28111824 PMC5412922

[17] Lin G Zhuang C Shen Z Xiao G Chen Y Shen Y. APT Weighted MRI as an effective imaging protocol to predict clinical outcome after acute ischemic stroke. Front Neurol. (2018) 9:901. 10.3389/fneur.2018.00901 30405523 PMC6205981

[18] By S Barry RL Smith AK Lyttle BD Box BA Bagnato FR. Amide proton transfer CEST of the cervical spinal cord in multiple sclerosis patients at 3T. Magn Reson Med. (2018) 79:806–14. 10.1002/mrm.26736 28474409 PMC5777527

[19] Dula AN Asche EM Landman BA Welch EB Pawate S Sriram S. Development of chemical exchange saturation transfer at 7 T. Magn Reson Med. (2011) 66:831–8. 10.1002/mrm.22862 21432902 PMC3156337

[20] Zhou J Heo HY Knutsson L van Zijl PCM Jiang S. APT-Weighted MRI: techniques, current neuro applications, and challenging issues. J Magn Reson Imaging. (2019) 50:347–64. 10.1002/jmri.26645 30663162 PMC6625919

[21] Heo HY Zhang Y Burton TM Jiang S Zhao Y van Zijl PCM. Improving the detection sensitivity of pH-weighted amide proton transfer MRI in acute stroke patients using extrapolated semisolid magnetization transfer reference signals. Magn Reson Med. (2017) 78:871–80. 10.1002/mrm.26799 28639301 PMC5561454

[22] Zhou IY Lu D Ji Y Wu L Wang E Cheung JS. Determination of multipool contributions to endogenous amide proton transfer effects in global ischemia with high spectral resolution *in vivo* chemical exchange saturation transfer MRI. Magn Reson Med. (2019) 81:645–52. 10.1002/mrm.27385 30058148 PMC6258351

[23] Mistry N Abdel-Fahim R Samaraweera A Mougin O Tallantyre E Tench C. Imaging central veins in brain lesions with 3-T T2^*^-weighted magnetic resonance imaging differentiates multiple sclerosis from microangiopathic brain lesions. Mult Scler. (2016) 22:1289–96. 10.1177/1352458515616700 26658816

[24] Reitz SC Hof SM Fleischer V Brodski A Gröger A Gracien RM. Multi-parametric quantitative MRI of normal appearing white matter in multiple sclerosis, and the effect of disease activity on T2. Brain Imaging Behav. (2017) 11:744–53. 10.1007/s11682-016-9550-5 27138529

[25] Van de Ven K Keupp J Amide Proton Transfer Weighted Imaging: Advancement in Molecular Tumor Diagnosis. 3D APT Whitepaper. Philips® Healthcare (2018).

[26] Kamimura K Nakajo M Yoneyama T Fukukura Y Hirano H Goto Y Histogram analysis of amide proton transfer-weighted imaging: comparison of glioblastoma and solitary brain metastasis in enhancing tumors and peritumoral regions. Eur Radiol. (2018) 28:4133–40. 10.1007/s00330-018-5832-1 30488111

[27] Obuchowski NA. Nonparametric analysis of clustered ROC curve data. Biometrics. (1997) 53:567–78. 10.2307/2533958 9192452

[28] Jiang S Rui Q Wang Y Heo HY Zou T Yu H. Discriminating MGMT promoter methylation status in patients with glioblastoma employing amide proton transfer-weighted MRI metrics. Eur Radiol. (2018) 28:2115–23. 10.1007/s00330-017-5182-4 29234914 PMC5884703

[29] Zhang Z Yip CY Grissom W Noll DC Boada FE Stenger VA. Reduction of transmitter B1 inhomogeneity with transmit SENSE slice-select pulses. Magn Reson Med. (2007) 57:842–7. 10.1002/mrm.21221 17457863 PMC3041897

[30] Sun PZ Farrar CT Sorensen AG Correction for artifacts induced by B0 and B1 field. Magn Reson Med. (2007) 58:1207–15. 10.1002/mrm.21398 17969015

[31] Zu Z. Towards the complex dependence of MTRasym on T1w in amide proton transfer (APT) imaging. NMR Biomed. (2018) 31:e3934. 10.1002/nbm.3934 29806717 PMC6089235

[32] Zhang XY Wang F Li H Xu J Gochberg DF Gore JC. Accuracy in the quantification of chemical exchange saturation transfer (CEST) and relayed nuclear Overhauser enhancement (rNOE) saturation transfer effects. NMR Biomed. (2017) 30:e3716. 10.1002/nbm.3716 28272761 PMC5490367

[33] Heo HY Lee DH Zhang Y Zhao X Jiang S Chen M. Insight into the quantitative metrics of chemical exchange saturation transfer (CEST) imaging. Magn Reson Med. (2017) 77:1853–65. 10.1002/mrm.26264 27170222 PMC5107181

[34] Sartoretti T Sartoretti E Wyss M Schwenk Á Najafi A Binkert C. Amide proton transfer contrast distribution in different brain regions in young healthy subjects. Front Neurosci. (2019) 13:520. 10.3389/fnins.2019.00520 31178687 PMC6538817

[35] Zhao X Wen Z Huang F Lu S Wang X Hu S. Saturation power dependence of amide proton transfer image contrasts in human brain tumors and strokes at 3 T. Magn Reson Med. (2011) 66:1033–41. 10.1002/mrm.22891 21394783 PMC3136645

[36] Tietze A Blicher J Mikkelsen IK Østergaard L Strother MK Smith SA. Assessment of ischemic penumbra in patients with hyperacute stroke using amide proton transfer (APT) chemical exchange saturation transfer (CEST) MRI. NMR Biomed. (2014) 27:163–74. 10.1002/nbm.3048 24288260 PMC4019439

[37] Tee YK Harston GW Blockley N Okell TW Levman J Sheerin F. Comparing different analysis methods for quantifying the MRI amide proton transfer (APT) effect in hyperacute stroke patients. NMR Biomed. (2014) 27:1019–29. 10.1002/nbm.3147 24913989 PMC4737232

[38] Yu L Chen Y Chen M Luo X Jiang S Zhang Y. Amide proton transfer MRI signal as a surrogate biomarker of ischemic stroke recovery in patients with supportive treatment. Front Neurol. (2019) 10:104. 10.3389/fneur.2019.00104 30853932 PMC6395437

[39] Song G Li C Luo X Zhao X Zhang S Zhang Y. Evolution of cerebral ischemia assessed by amide proton transfer-weighted MRI. Front Neurol. (2017) 8:67. 10.3389/fneur.2017.00067 28303115 PMC5332413

[40] Duchesnay E Hadj Selem F De Guio F Dubois M Mangin J-F Duering M Ropele S Schmidt R. Different types of white matter hyperintensities in CADASIL. Front Neurol. (2018) 9:526. 10.3389/fneur.2018.00526 30042721 PMC6048276

[41] Schirmer MD Giese A-K Fotiadis P Etherton MR Cloonan L Viswanathan A. Spatial signature of white matter hyperintensities in stroke patients. Front Neurol. (2019) 10:208. 10.3389/fneur.2019.00208 30941083 PMC6433778

[42] Frey BM Petersen M Mayer C Schulz M Cheng B Thomalla G. Characterization of white matter hyperintensities in large-scale MRI-studies. Front Neurol. (2019) 10:238. 10.3389/fneur.2019.00238 30972001 PMC6443932

[43] Muñoz Maniega S Meijboom R Chappell FM Valdés Hernández MdC Starr JM Bastin ME. Spatial gradient of microstructural changes in normal-appearing white matter in tracts affected by white matter hyperintensities in older age. Front Neurol. (2019) 10:784. 10.3389/fneur.2019.00784 31404147 PMC6673707

[44] Li Y Li M Zuo L Shi Q Qin W Yang L. Compromised blood–brain barrier integrity is associated with total magnetic resonance imaging burden of cerebral small vessel disease. Front Neurol. (2018) 9:221. 10.3389/fneur.2018.00221 29681883 PMC5897516

[45] Wu X Ge X Du J Wang Y Sun Y Han X. Characterizing the penumbras of white matter hyperintensities and their associations with cognitive function in patients with subcortical vascular mild cognitive impairment. Front Neurol. (2019) 10:348. 10.3389/fneur.2019.00348 31031687 PMC6474292

